# Growth Inhibition and Apoptotic Effect of Pine Extract and Abietic Acid on MCF-7 Breast Cancer Cells via Alteration of Multiple Gene Expressions Using In Vitro Approach

**DOI:** 10.3390/molecules27010293

**Published:** 2022-01-04

**Authors:** Hesham Haffez, Shimaa Osman, Hassan Y. Ebrahim, Zeinab A. Hassan

**Affiliations:** 1 Biochemistry and Molecular Biology Department, Faculty of Pharmacy, Helwan University, Cairo 11795, Egypt; zihassan2014@gmail.com; 2 Helwan Structural Biology Center for Excellence, Helwan University, Cairo 11795, Egypt; 3 Helwan General Hospital, Cairo 11731, Egypt; shaimaa_abdelrheem@hotmail.com; 4 Pharmacognosy Department, Faculty of Pharmacy, Helwan University, Cairo 11795, Egypt; hebrahim@pharm.helwan.edu.eg

**Keywords:** abietic aid, P. palustris, anti-cancer, MCF-7, apoptosis

## Abstract

In vitro anti-proliferative activity of *Pinus palustris* extract and its purified abietic acid was assessed against different human cancer cell lines (HepG-2, MCF-7 and HCT-116) compared to normal WI-38 cell line. Abietic acid showed more promising IC_50_ values against MCF-7 cells than pine extract (0.06 µg/mL and 0.11 µM, respectively), with insignificant cytotoxicity toward normal fibroblast WI-38 cells. Abietic acid triggered both G_2_/M cell arrest and subG_0_-G_1_ subpopulation in MCF-7, compared to SubG_0_-G_1_ subpopulation arrest only for the extract. It also induced overexpression of key apoptotic genes (*Fas, FasL, Casp3, Casp8, Cyt-C* and *Bax*) and downregulation of both proliferation (*VEGF, IGFR1, TGF-β*) and oncogenic (*C-myc* and *NF-κB*) genes. Additionally, abietic acid induced overexpression of cytochrome-C protein. Furthermore, it increased levels of total antioxidants to diminish carcinogenesis and chemotherapy resistance. *P. palustris* is a valuable source of active abietic acid, an antiproliferative agent to MCF-7 cells through induction of apoptosis with promising future anticancer agency in breast cancer therapy.

## 1. Introduction

Natural products (also known as secondary metabolites) were proven as a prolific resource for the discovery of bioactive hit and lead compounds in the early stages of drug discovery. Secondary metabolites are distinguished by being novel in terms of chemical structures and biological activities [1]. In principle, natural products frequently encompass three-dimensional scaffolds and various chiral centers that inevitably mediate their selective interactions with disease-relevant macromolecular targets. In the anticancer drug discovery pipeline, natural products are one of the most robust starting points for initiating the development of anticancer therapeutics. This was demonstrated by the remarkable clinical successes of paclitaxel, doxorubicin, vincristine, vinblastine, and many other eminent examples [2]. Additionally, natural product-derived anticancer agents have the advantage of being less toxic to normal cells than malignant ones. This should surely provide some impetus to uncover the biological effects of natural products and study their interactions with cancerous cells at the molecular level in order to better understand cancer biology and hypothesize rational strategies for controlling cancers.

According to some ethnobotanical literature data, *Pinus* species have been used in folk medicine in Algeria [3] and Turkey [4], as well as many Islamic [5] (and many other) countries in the Cupisnique age, whether as analgesics [4], wound healers [4], or for their anti-inflammatory properties [4]. Pine-derived oleoresins comprise multiple varieties of terpenoids; they are the largest group of plant secondary metabolites with more than 30,000 known derivatives [6]. Terpenoids are biosynthesized from five-carbon isoprene units—namely, isopentyl diphosphate (IPP) and diallyldiphospahte (DPP)—via head-to-tail condensation catalyzed by prenyl transferase groups of enzymes in plant cells. Two units of IPP and DPP yield the basic cyclic geranyl diphosphate (GPP), which then condenses with another molecule of GPP to yield geranyl geranyl diphosphate (GGPP), the precursor of C20 diterpenes. After the construction of GGPP, a series of cyclization (via terpene synthases) and oxidation reactions occur in order to furnish various scaffolds. Diterpenoids are considered among the most ecologically useful traits in conifers, as they provide defense against pests and their associated pathogenic fungi [7]. As denoted from the name, pine oleoresin comprises a resin portion, known as rosin, and a volatile oil, known as turpentine. It has been proposed that turpentine oil functions as a solvent to facilitate the mobilization of resin constituents to injury sites. Meanwhile, rosin comprises various diterpene acids (known also as resin acids) and functions as an antipathogenic agent [8]. Resin acids have attracted the interest biologists, leading them to identify their biological effects and to elucidate their structure–activity relationships. Pimarane and abietane tricyclic scaffolds are the most abundant diterpenes in pine species. They have distinct biological activities as well as versatile applications in agriculture and medicine [9,10,11].

Abietic acid is a diterpenoid acid that has been isolated from different species of coniferous plants belonging to the genus *Pinus*, such as *P. palustris*, *P. insularis*, and *P. sylvesteris*. Diverse biological activities have been reported for abietic acid and its analogs, including anti-inflammatory [12,13], anticonvulsant [14], anti-obesity [15], cytotoxic [16], antimycotic [16,17], and antiviral activities [18]. Such bioactivities are correlated to specific interactions with relevant disease signaling mediators. For instance, the anti-inflammatory effect of abietic acid was proven to be mediated via attenuation of interleukin-1 beta in induced inflammatory models of human chondrocytes [13]. Furthermore, abietic acid was shown to inhibit multidrug resistance-associated protein 2 (MRP2)- and breast cancer resistance protein (BCRP)-mediated membrane transports and their interactions with substrates [19]. Additionally, a recent study by Lui et al. reported the remarkable antiproliferative effect of abietic acid on a panel of six non-small-cell lung cancer (NSCLC) cell lines [20]. However, there remains a need to identify the illustrated mechanisms in order to highlight abietic acid’s anti-cancer activities in different cancer cell lines in comparison with previously known apoptotic agents, such as Raptinal [21], and chemotherapeutic agent, such as Doxorubicin [22]. This study evaluated the biological activity of *Pinus palustris* extract, as well as its main constituent, abietic acid, for anti-proliferation and cytotoxic activity in different cancer cell lines with comparison to known anticancer agents. Moreover, the study shed light on different mechanistic pathways that may be modulated upon treatment of abietic acid to the most sensitive cancer cell lines. The results of this study may guide scientists to deepen our understanding of the mechanistic role of abietic acid—as well as future therapeutic optimizations and applications in in vivo and preclinical phases.

## 2. Results

### 2.1. Abietic Acid Induces Growth Inhibition in MCF-7 Sensitive Cell Line

In the current study, MTT assay was used to assess the antiproliferative activity of both pine extract and its pure abietic acid active compound against three different cancer cell lines. These were also compared to normal human fibroblast cell lines. Two positive controls of documented antiproliferative activities were used for comparison (Raptinal [21] and Doxorubicin [22]). The IC_50_ was calculated according to three parameters and the logistic dose-response sigmoidal curve using GraphPad Prism 4.0 (Figure S1). Table 1 shows positive controls, pine extract, and abietic acid with variable antiproliferative activities against the different cancer cell lines (HCT-116, HepG-2 and MCF-7). In HepG-2 hepatocellular carcinoma cells, Raptinal and Doxorubicin showed antiproliferative activity with IC_50_ = 0.74 and 0.8 µM, respectively, which was similar to the documented values in the literature [23]. Pine extract showed superior antiproliferative activity with IC_50_ = 0.2µg/mL. 

The selectivity index (SI) is defined as the ratio of the toxic concentration of a sample against its therapeutic concentration [24]. The ideal drug for continuation in drug screening should have a relatively high toxic concentration in order to cause toxicity at very low doses for therapeutic effect. Candidate drug should have generally SI value > 1 and fall into one of the following criteria: SI value = 1–1.99 is of low selectivity (general toxicity), SI value = 2–2.99 is of moderate selectivity and SI value >3 is of high selectivity [25,26,27,28,29,30]. Therefore, evaluation of SI values for any herbal drug and/or isolated compound is crucial for determining whether further work can be done. Pine extract’s SI value was 2, indicating less cytotoxic effects than Raptinal (SI = 7). Abietic acid did not show any significant antiproliferative effects on HepG-2 cells. The same scenario was observed in HCT-116 human colon cancer cells, where pine extract showed superior antiproliferative activity, with IC_50_= 0.25 µg/mL, and minimal cytotoxic effects, with SI = 1.7, as compared to the nonsignificant effects of abietic acid. MCF-7 was the most sensitive cell line, with minimum cytotoxicity for both pine extract and its pure isolated abietic acid (IC_50_ = 0.11µg/mL, SI = 3.8 and 0.06 µM, SI > 1, respectively (Table 1). SI > 1 for abietic acid indicated that it was the safest, since very high concentrations, i.e., above 100 µM, were required for cytotoxic effects on WI-38, as compared to IC_50_ on MCF-7 cells. The IC_50_ value for abietic acid was not applicable on WI-38, HepG-2, or HCT-116, as we tested several x-fold concentrations and dilutions of abietic acid on both extremities without 50% cellular viability inhibition. Therefore, there was no need to perform further experiments with them on these cell lines; there was almost no chance for them to be used as therapeutic drugs on these cell lines. Any dose above 100 µM or 0.5 mg/mL is considered within cytotoxic margins, as documented by many studies [31,32,33,34,35] and, consequently, calculation of SI value was not applicable.

### 2.2. Abietic Acid Induces MCF-7 Cells Apoptosis with Cell Cycle Arrests at Both G0-G1 and G2/M Phases and SubG0-G1 Sub-Population

**Figure 1:**
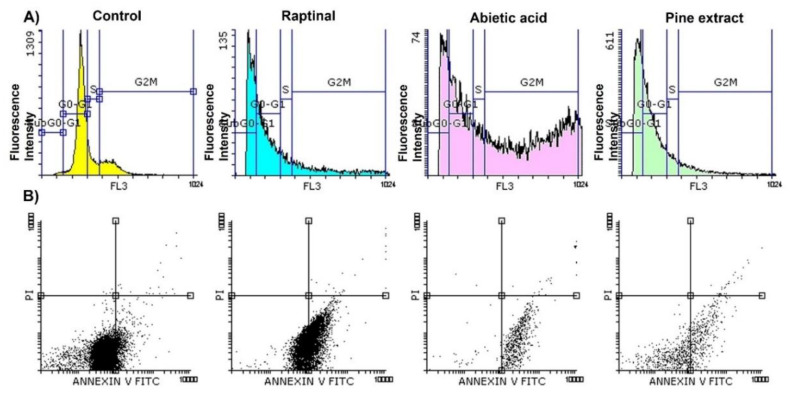
Flow cytometry analysis of treated MCF-7 cells over 24 h with 0.1% DMSO-treated cells as control, IC_50_ of pine extract, abietic acid, and Raptinal; (**A**) Cell cycle analysis showing negative control exposed to 0.1% DMSO solvent with a higher percentage of dividing cells in G_0_-G_1_ phase (yellow), Raptinal with higher cell arrest in G_0_-G_1_ phase (magenta), abietic acid with cell cycle arrest in G_2_/M phase (pink) and subG_0_-G_1_ subpopulation, pine extract with cell arrest in SubG_0_-G_1_ subpopulation (green). Pine extract induced SubG_0_-G_1_ subpopulation arrest, while abietic acid induced both G_2_/M cell phase and SubG_0_-G_1_ subpopulation arrest. (**B**) Apoptotic analysis using MCF-7 cells for negative control cells, Raptinal, abietic acid, and pine extract. Dot plots show early apoptotic dead cells with abietic acid and pine extract in the right bottom quadrant.

The SubG0-G1 subpopulation is an important fraction of the nondividing cells that tend to enter cell cycle division. Hence, compounds that are able to induce cell cycle accumulation in this subpopulation have a powerful apoptotic effect and inhibit scell growth at early stage [36,37,38]. Since abietic acid showed potent antiproliferation activity against MCF-7 cells, the next step was to investigate its apoptotic and cell cycle effects in comparison to pine extract on MCF-7 cell cycle. MCF-7 cells were incubated with IC_50_ of abietic acid and pine extract for 24 h and flow cytometry assay was undertaken. The cell cycle phases, and subpopulation were set in gate, as previously documented [39]. The data in Figure 1A, Table 2 and Table 3 show that the number of cells arrested in the SubG_0_-G_1_ subpopulation increased significantly for cells treated with both abietic acid and pine extract in a way similar to known apoptotic positive controls of Raptinal-treated cells. Consequently, the gated G0-G1 region was reduced significantly due to the massive arrest in SubG_0_-G_1_ subpopulation after both pine extract and abietic acid treatment compared to control, with 42.07% and 24.98%, respectively, compared to 66.59% of control cells treated with 0.1% DMSO solvent, as shown in Table 2. Moreover, abietic acid showed additional cell cycle arrest for cells at G_2_/M phase, which was not observed in pine extract, with 49.17% and 11.34%, respectively, compared to control at 18.73% and Raptinal at 18.63%. This was reflected in the G_2_M/G_0_-G_1_ ratio, which was <1 (0.71) for negative control, Raptinal, and pine extract. Meanwhile, abietic acid had a significantly higher G_2_M/G_0_-G_1_ ratio, 2-fold higher than pine extract, suggesting that abietic acid was reducing the percentage of dividing cell populations in G_2_/M phase.

To quantify the cell populations in each apoptotic stage, we employed the Annexin V-FITC/PI double staining assay using a flow cytometer. As shown in Figure 1B, the early apoptotic cells were positive for Annexin V (the lower right quadrant). The late and necrotic cell populations are shown in the upper right and left quadrants, respectively. The data demonstrated that the amount of early apoptosis of MCF-7 cells was raised after exposure to both pine extract and abietic acid, as compared to control, after 24 h treatment. As shown in Table 3, IC_50_ of abietic acid and pine extract raised apoptosis from 6.3% for control cells treated with 0.1% DMSO to 91.41% and 71.39%, respectively, with superior activity for abietic acid (even higher than Raptinal at 51.35%). Apoptotic index (AI) is the marker for apoptotic efficacy and can be calculated by dividing the percentage of early apoptotic cells by the total percentage of viable and early apoptotic cells with AI = 1 or higher indicating high efficacy. In control, Raptinal, and pine extract, AI was <1, while abietic acid had AI = 1. This supported the previously observed apoptotic efficacy of abietic acid over pine extract.

### 2.3. Abietic Acid Modulates Key- Genes Regulating Multiple Controlling Pathways

It was essential to understand the possible molecular mechanisms regulating apoptosis that occurred after treatment of MCF-7 with both pine extract and abietic acid on the level of genetic expression in order to predict possible mechanistic changes at the early stage. Thus, it was of particular interest to investigate the expression pattern of a group of apoptosis-related genes (extrinsic and intrinsic pathways) over different time scales—4, 8, and 24 h. The treatment periods chosen for the analysis of abietic acid action were based on previous studies in the same area [40,41]. Table 4 shows the gene expression levels of different genes of different pathways. Both pine extract and abietic acid were able to induce gene overexpression of extrinsic apoptotic driven genes, such as *Fas, FasL* and *Casp8*, in all selected time scales, with maximum peaks between 4 and 8 h.

However, abietic acid showed markedly significant increases in gene expression levels after 24 h of *Cyto-C, Bax*, and *p53*, which play important roles in intrinsic pathways with lower levels of *Bcl-2* and a higher *Bax/Bcl-2* ratio. Furthermore, *p53*-mediated transactivation of apoptosis comes from its ability to control transcription of proapoptotic members of the *Bcl-2* family. Additionally, *p53* activation is able to activate other caspases, a cascade that consequently activates *Cyto-C* and apoptosome formation [42]. *Bax* genes and *Bcl-2* were expressed in harmonic rhythm, wherein the *Bax* gene was overexpressed to a maximum peak after 24 h, while the *Bcl-2* gene was downregulated in the same ratio. Additionally, the *Bax/Bcl-2* ratio, which acts as a rheostat and determines cell susceptibility to apoptosis [43,44], reached its maximum (13.9) with only abietic acid treatment after 24 h.

Finally, the *p53* gene did not show any genetic modification after treatment with pine extract, while it was overexpressed after 24 h with abietic acid. The *ATG5* gene is an important interplay mediator and was shown to overexpressed with both pine extract and abietic acid after 24 h and to play a dual role in both autophagy and apoptosis. *BNIP3* is the network mediator connecting the two types of cell death pathways; it was also over-expressed after 8 h. This may suggest that both pine extract and abietic acid activities were achieved within 4–8 h of MCF-7 treatment, and maximum gene modifications were observed after 24 h.

The expression of proliferation target genes such as *VEGF, TGF-β1, IGF1R* and *ATG12*, and levels of oncogenes *C-myc, TNF-α* and *NF-κB* were downregulated over all time scales and did not change significantly. This may suggest the potential role of both pine extract and abietic acid in apoptosis through angiogenesis and carcinogenesis.

It was noted that kinases play important roles as molecular therapeutic targets in modern anticancer therapy. Pine extract and abietic acid were shown to downregulate the level of *PKC-α* after 4 h, with superior significant reduction for abietic acid-treated cells. This effect lasted at a minimum level after 24 h. Another key kinase in MCF-7 resistance and metastasis is *PRKAA1* (Protein Kinase AMP-Activated Catalytic Subunit Alpha 1); both pine extract and abietic acid were able to overexpress *PRKAA1*, which activated AMPK after 4 h and increased in a time-dependent manner after 24 h. Finally, pine extract was shown to slightly induce the overexpression of *CDK-4* after 8 h, but downregulation occurred after 24 h, while abietic acid reduced *CDK-4* gene levels after 24 h.

### 2.4. Abietic Acid Induces Increased Protein Level of Cytochrome-C Confirmed by Immunocytochemistry (ICC) Analysis

**Figure 2:**
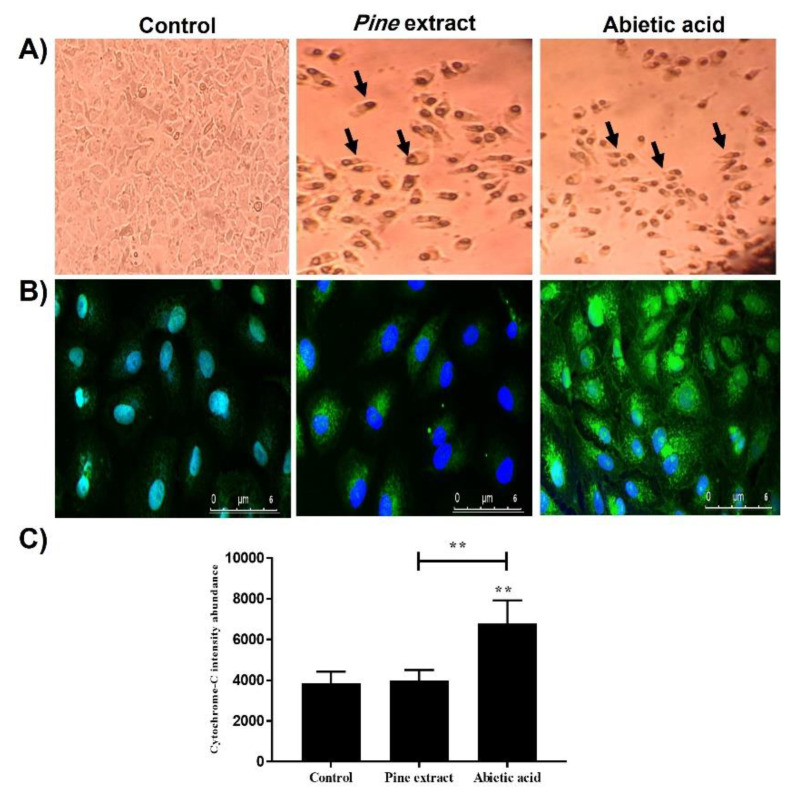
Cell morphology and immunocytochemistry of MCF-7 treated with IC_50_ of pine extract or abietic acid for 24 h and stained with anti-cytochrome-C; (**A**) MCF-7 cells treated with 0.1% DMSO compared to effects of pine extract and abietic acid under an inverted microscope (OPTECH Biostar IB, magnification, ×40). Arrows indicate dark rounded apoptotic bodies. (**B**) Comparative study for anti-cytochrome-C staining of control cells compared to cells treated with either pine extract or abietic acid and (**C**) significant increase in cytochrome-C protein expression level demonstrated with abietic acid-treated cells, as compared to both control and pine extract. The values are considered statistically significant to untreated control at ** p < 0.01. Results are expressed as the mean ± SEM (*n* = 3).

The apoptotic effect was morphologically confirmed, as shown in Figure 2A. Cells treated with IC_50_ of pine extract and abietic acid showed similar characteristic features of cellular apoptosis, with some cells detached and floating in cell culture media. In addition, shrinkage of the MCF-7 cells was observed, with dark rounded apoptotic bodies, as shown, with both total extract- and abietic acid-treated cells compared to control, 0.1% DMSO-treated cells. Using ICC analysis, cytochrome-C release and overexpression was shown with both MCF-7 cells treated with pine extract and abietic acid, compared to control cells (Figure 2B). However, the expression level was significantly higher with abietic acid compared to control and pine extract-treated cells (Figure 2C).

### 2.5. Total Antioxidant Assay

**Figure 3:**
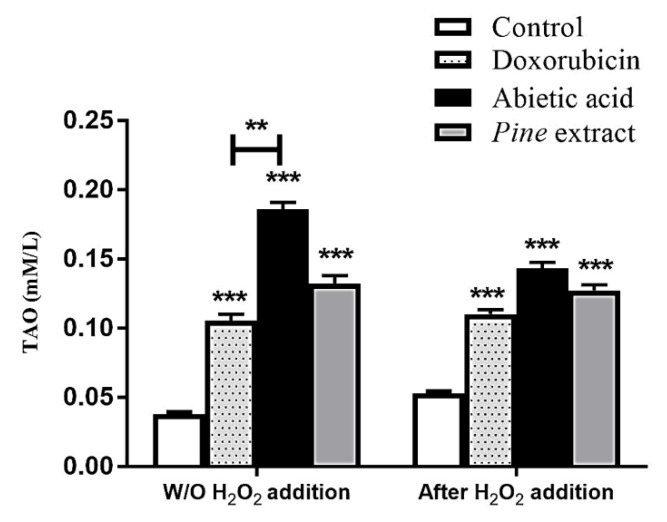
Total antioxidant activity (TOA) of IC_50_ dose of both pine extract and abietic acid treatment of MCF-7 cells for 24 h. Both extract and abietic acid induce significant increases in total antioxidant capacity as compared to negative control without (W/O) H_2_O_2_. TOA calculated compared to negative control only (in case of without H_2_O_2_), or treated with 50 µM hydrogen peroxide for 1 h. Data represented as TOA ± SEM, *n* = 3. Results are presented as ± SEM, *p* value, for comparison with Doxorubicin control, is ** p < 0.01, *** p < 0.001.

It was essential to screen the potential antioxidant power of pine extract and abietic aid in order to diminish the endogenous oxidative stress in MCF-7 cells and to counteract free radical overproduction after exposure to low levels of hydrogen peroxide. Data presented in Figure 3 show that control MCF-7 cells treated with 0.1% DMSO secreted 0.04 mM of total antioxidants (TAO). Both pine extract and abietic acid treatment with IC_50_ for 24 h showed significantly increasing TAO, to 0.14 and 0.19 mM/L, respectively, compared to control cells treated with 0.1% DMSO solvent. Abietic acid showed additional significant increases in TAO levels compared to Doxorubicin-treated cells (0.11 mM/L). We observed that abietic acid induced significantly higher levels of TAO compared to both Doxorubicin and pine extract. Moreover, exposure of MCF-7 cells to 50 µM of H_2_O_2_ for 1 h as inducer for intrinsic low levels of oxidative stress, led to slightly increased TAO of control cells (to 0.05 mM). Treatment after H_2_O_2_ with either IC_50_ of pine extract or abietic acid induced significantly higher TAO levels (0.13 and 0.14 mM/L, respectively) compared to control treated with H_2_O_2_ and Doxorubicin-treated cells (0.11 mM/L). However, there was no significant difference in TAO with or without treatment with H_2_O_2_. This might have been due to the significant apoptosis induced with the pine extract and abietic acid and the associated reduced TAO content.

## 3. Discussion

Although conventional anticancer drugs exhibit high efficacy against different types of cancer, obvious side effects have been observed. Additionally, the resistance of cancer cells to these drugs as a consequence of cell mutations is considered an obstacle and an enormous crisis in terms of increasing morbidity and mortality rates. Consequently, multi-targeted approaches using medicinal plants and phytoconstituents in combination with synthetic drugs are becoming an effective method to overcome these limitations. Pine species are an interesting example of widespread medicinal plants, with over one hundred species worldwide and a long track record of medicinal uses. Pine species’ needles, inner bark, and resin are rich with many natural compounds possessing potent biological activities, such as abietic acid [45]. Additionally, pine remedies have been proven effective as anti-infection therapies for urinary tract infections, sinus infections, and lung-associated illnesses or allergies such as coughs and colds [46]. Topically, pine has been reported as anti-inflammatory agent with arthritis and skin infections [47]. Abietic acid is the major constituent of *P. palustris* from the resin acid [48]. In vitro, abietic acid has been shown to function as a testosterone 5α-reductase inhibitor and can be used for treatment of benign tumors such as prostatic hyperplasia [49]. It was interesting to study its in vitro effects in comparison to pine extract in order to understand its potential anticancer activity and cytotoxicity against different cancer cell lines and normal cells.

The antiproliferation and cytotoxicity results showed that the isolated abietic acid is more potent, compared to pine extract, against breast cancer cell lines (MCF-7), with minimal cytotoxicity to normal cells. This observation matched recent studies that have shed light on the ability of pine extracts and pure abietic acid prepared from various pine species to exert anticancer effects [50,51,52,53]. However, data presented in this study are the first, to our knowledge, to demonstrate the ability IC_50_ of pine extract and abietic acid to suppress the viability of human breast cancer MCF-7 cells, and the first study to elucidate the mechanism of their action. MCF-7 was put to use as the sensitive cell line and tested with both pine extract and abietic acid for further analysis, to demonstrate its possible anticancer activity and potency. These may be developed as candidates for chemoprevention or as a chemotherapeutic adjuvant for breast tumors.

Cell cycle analysis showed abietic acid increased cell population arrest exclusively in the G_2_/M phase as well as the subG0-G_1_ subpopulation of breast cancer cell cycle, suggesting that abietic acid-induced signaling leads to breast cancer cell antiproliferation [50,54]. Indeed, our live/dead assay (apoptosis assay) confirmed that abietic acid was able to induce early apoptosis in a way similar to pine extract, as indicated by higher Annexin-V staining than propidium iodide in MCF-7 treated cells relative to the control. The combination of different bioactive ingredients in the extract may explain the difference seen between pine extract and abietic acid affecting cells in cell cycle phases [55]. Still, abietic acid showed more significant apoptotic effects on MCF-7 than pine extract, since it was additionally able to induce MCF-7 cell cycle arrest in G_2_/M phase (more so than Raptinal, a standard apoptotic agent). This observation suggested that pure isolated abietic acid could have more specific effects on cell cycles and may induce cell population shifts in different phases due to its standardization, thereby avoiding endo-interactions with other bioactive ingredients. Hence, the purity potentiates its apoptotic effects, as opposed to the complex nature of the pine extract [55,56,57,58,59,60,61]. Our data concurred with previously reported results showing that abietic acid showed antitumor activity through apoptosis induction and alteration of apoptosis-related proteins (Bax, caspase 3 and 9) in nasopharyngeal carcinoma (NPC) and triggered cell cycle arrest at G_2_/M phase [50]. Taken together, the presented results demonstrated that pine extract and pure isolated abietic acid were able to induce MCF-7 cell apoptosis with potential differences in activity.

These findings were correlated at the molecular level while exploring the modulation of different key-signaling genes controlling apoptosis, resistance, carcinogenesis, vascularization, and cellular growth. Abietic acid was significantly able to induce an increase in both key intrinsic and extrinsic apoptosis genes, such as (*Fas, FasL, BNIP3, Casp8, Cyto-C, Bax*). This suggested that MCF-7 responded very well to the apoptotic genetic changes within 24 h of treatment. However, abietic acid treatment was able, after a short time (8–24 h), to render cells reliable for diminished resistance, since *Bax* and counteracting twin *Bcl-2* family genes are good prognostic markers for cellular progression and aggressiveness and their levels were changed after treatment [62,63].

After 8 h of *Bax* overexpression and downregulation of *Bcl-2* genes, *Bax* transcription and induction induced stress-activated *p53* apoptosis on the mitochondrial membrane which, consequently, overcame the antiapoptotic effects of *Bcl-2*. Thus, abietic acid might be able to induce *p53*-dependent apoptosis, after 24 h, that is attenuated in the presence of *Bax* with diminished *Bcl-2*. Some reported studies demonstrated this correlation between expression of *Bax* and *p53* genes [64]. Hence, the ratio of *Bax* to *Bcl-2* gene levels could influence the fate of a cell in response to apoptosis by abietic acid. This conclusion supported the enhanced activity of abietic acid in apoptosis, as compared to pine extract. Another gene marker is *ATG5*, which is known for its potential role in controlling apoptosis/autophagy. *ATG5* overexpression was evident after abietic acid treatment concurrently with *Fas* and *FasL* genes after 24 h, suggesting its previously reported role in apoptosis through interaction with *FADD* (*Fas*-associated protein with death domain), and suggesting that this interaction mediates interferon-γ (IFN-γ)-induced cell death [65]. Moreover, abietic acid was able to induce downregulation of the key gene controlling vascularity and blood supply to cancer cells such as *VEGF* and, hence, vascular permeability, angiogenesis, proliferation, and cell resistance mediated through several cancer-causing factors [66]. Furthermore, abietic acid was shown to inhibit the expression of *c-myc*, an oncogene that belongs to a family of genes that potentially regulate transcription on the genome level. Previous studies showed that its downregulation enhanced the effects of anticancer therapies on cell cycle arrest, apoptosis, and the invasion and migration of different cancer cell in vitro [67]. This effect could be explained by regulating the HIF-1α/SDF-1/CXCR4 signaling pathway [68]. Another marker deemed essential in cancer resistance is *TNF-α*, which is a proinflammatory cytokine that exaggerates cellular inflammation, proliferation, and carcinogenesis in MCF-7 cells [69]. The activation of NF-κB signaling, induced by *TNF-α*, has also been shown to be a key element in resistance of apoptosis-based tumor mechanisms. Thus, it is an attractive target for modulation by abietic acid in therapy in order to facilitate the enhancement of *TNF*-*α*-mediated apoptosis for anticancer treatment in MCF-7 cells [20,69]. These results could support the potency of pine extract and abietic acid in any combined adjuvant remedy with anticancer therapies due to the inhibition of *TNF-α*-induced cancer cell invasion and angiogenesis.

Abietic acid also showed modulating effects on some kinases’ expression, such that their activity played a key role in controlling different carcinogenesis and malignant transformation pathways through ATP regulation and enhancement of anticancer sensitivity and cellular responses [70]. Moreover, dysregulation of kinases is considered an oncogenic prognostic marker for apoptotic resistance and spread of cancer cells [70]. For example, higher levels of *PKC-α* genes in MCF-7 have been associated with higher proliferation rates and transformation of epithelioid morphological appearances into tumors in nude mice [71]. MCF-7-inducing higher *PKC-α* genes exhibited significant reductions in estrogen receptor expression and decreases in estrogen-dependent gene expression [71]. These findings suggested that the inhibition of the *PKC* pathway could modulate the progression of breast cancer to a more aggressive neoplastic process, which was observed in our study, as well. Abietic acid and pine extract were able to inhibit the growth of *PKC-α.* Protein Kinase AMP-Activated Catalytic Subunit Alpha 1 (*PRKAA1*) is another regulating factor in carcinogenesis [72]. Cancer growth and metastasis is a result of imbalances between energy-producing systems and consumption of the energy for growth. AMP-activated protein kinase (*AMPK*) has been shown to regulate this process through regulation of AMP and ADP levels. [72]. In carcinogenesis, AMPK signaling is inhibited and cells produce energy for growth and motility, opposing the actions of insulin and growth factors. Increasing AMPK activity could prevent the proliferation and metastasis of tumor cells [72]. In addition, AMPK suppresses aromatase, which is responsible for production of estrogen and breast cancer growth [72]. Thus, inhibition of AMPK is a good target in anticancer therapy. Our observed data coincided with previously noted studies and could provide additional approaches for the use of abietic acid in the prevention of breast cancer neoplasia, growth, and metastasis. Cyclin-dependent kinase 4 (*CDK-4*) was shown, in MCF-7, to lead into uncontrolled cell division, downstream of many mitogenic signaling pathways. This has implications for resistance [73]. Many CDK-4 inhibitors were recently discovered and shown to be effective inhibitors of MCF-7 poor prognosis and resistance [74]. Both pine extract and abietic acid were able to suppress expression levels of the *CDK-4* gene after 24 h.

It was essential, in this study, to investigate the role of the activation of all the previously mentioned pro- and apoptotic genes in activating the final executioner protein of apoptosis on the mitochondrial outer membrane (MOM). MOM plays a key role in intrinsic apoptosis, exaggerated by permeabilization and cytochrome c release to the cytoplasm [75]. Cytochrome-C release is activated by several proapoptotic stimuli, such as *Bax* and *Bcl-2* gene levels and, once activated, it triggers the activation of a class of protease enzymes called caspases that further activate the formation of apoptosome. Thus, investigating protein levels of cytochrome-C is of substantial importance to confirm the activation of pro-apoptotic (*Bax/Bcl-2*) and apoptotic (*Casp-3* and *casp-8*) genes and apoptosome formation. It has been suggested that the apoptotic effects of abietic acid and pine extract are mediated through intrinsic apoptosis. Therefore, it was essential to assess apoptotic changes after treatment on the protein level using cytochrome-C level [76]. There are many techniques for assessment of cytochrome-C release, such as western blotting and immunocytochemistry. However, western blotting had some limitations, including difficulty accurately determining the exact quantities of cytoplasmic cytochrome-C on X-ray film and whether those quantities exceeded the amount normally present within cytoplasm. Hence, immunocytochemistry was used in this study to assess the expression of cytochrome-C in both cytosolic and mitochondrial fractions. This was done confocal microscopy coupled with the sensitivity of an Alexa Fluor 488 for quantitative analysis [77].

**Figure 4:**
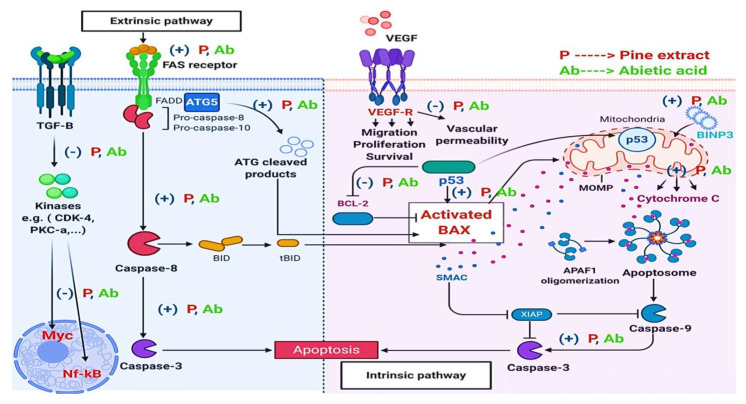
Summary of the suggested mechanistic effects of both pine extract and abietic acid on gene expression levels.

Cytosolic cytochrome-C activated further proteolytic activation of procaspase-9 to caspase-9 which, in turn, further activated caspase-3 with formation of apoptosome complex [78]. The cytosolic cytochrome-C can be linked also with previously observed gene expression levels of apoptotic genes, such as Fas and caspase-8, and extrinsic apoptosis, as described previously by others [79,80]. Thus, our ICC observed results could explain the potent ability of abietic acid to induce cell apoptosis by overexpression of the cytochrome-C gene through intrinsic dependent pathways. In summary, all the mechanistic actions of both pine extract and abietic acid are shown in Figure 4.

Cellular oxidative stress is important factor that reflects the inability of cells to remove intracellular reactive oxygen species (ROS) [81,82]. Although some ROS are beneficial for redox signaling pathways [83], certain levels of ROS are toxic to cells [84,85] and can react with all cellular components, e.g., proteins, lipids, and DNA, causing oxidative damage [86,87]. Moreover, ROS have been demonstrated to activate oncogenes, mutagenesis, and genomic variability in cancer cells and to stimulate cancer progression [88,89]. Recent studies have shown that cancer cells that have low ROS levels have augmented expression of ROS-scavenging signaling proteins and better responses to chemotherapy [84,88]. Abietic acid was able to induce overexpression of total antioxidants in MCF-7 in both resting state and under exogenous oxidative stress induced by H_2_O_2_.

## 4. Materials and Methods

### 4.1. Plant Material Preparation

#### 4.1.1. Plant Material and Resin Collection

Oleoresin was collected from a *Pinus palustris Mill. (Pinaceae)* tree growing at Helwan Agriculture Road, Cairo, Egypt, between May and August 2017. A voucher specimen # 01Ppa/2017 was deposited at the Herbarium of Pharmacognosy Department, Faculty of Pharmacy, Helwan University (Cairo, Egypt). The oleoresin exudate was scraped off, collected, and then subjected to stream distillation to remove turpentine oil. The remaining marc was dried at 60 °C under reduced pressure to yield a yellowish mass.

#### 4.1.2. Extraction and Purification of Abietic Acid

**Figure 5:**
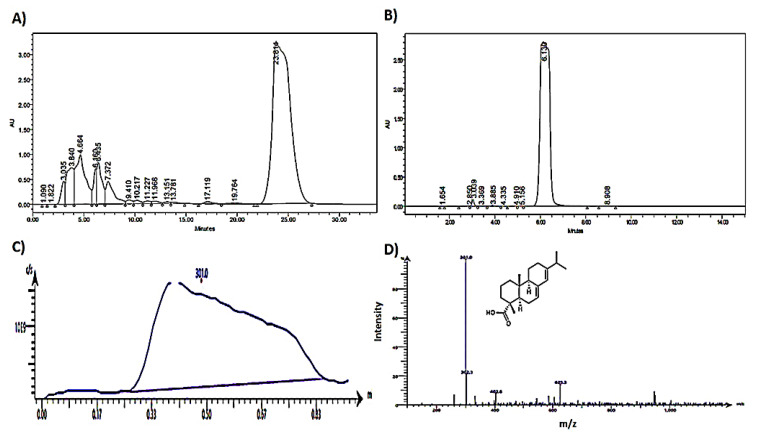
Analysis of purified abietic acid, (**A**) preparative HPLC chromatogram of abietic acid purification, (**B**) analytical HPLC chromatogram of purified abietic, (**C**) mass spec. analysis of the isolated abietic acid as total ion current of the injected sample, and (**D**) mass spec. analysis of the isolated abietic acid as negative-mode ESI mass spectrum of the injected sample.

About five grams of the dried residue, left after distilling the collected oleoresin, were extracted with 50 mL methanol at 50 °C under reflux. The concentrated alcoholic extract (2.7 g) was applied to silica gel open column (50 × 250 mm) and the elution was accomplished through stepwise gradient, starting with dichloromethane and gradually increasing the polarity by adding methanol (5% increments). Eluted fractions were monitored and collected based on their TLC patterns on precoated silica gel F254 plates (Merk, Darmstadt, Germany) using dichloromethane:methanol (9:1) as the developing system. Spots were detected under UV lamp (245 nm) and visualized by spraying with p-anisaldehyde/H_2_SO_4_ reagent followed by heating at 110 °C for 5 min for maximum color development. Fractions eluted by dichloromethane:methanol (9:1) were collected together and purified over multiple subcolumns of silica gel and finally subjected to purification by preparative HPLC. Approximately 40 mg of abietic acid rich fraction was injected into an Interchrom reversed-phase C-18 prep column (15 μm, 21.2 × 250 mm) installed on a Waters Alliance HPLC system (Agilent, Milford, MA, USA). The Empower^®^3 software package (Waters corporation, Milford, CT, USA) was used to control operation conditions and for spectral acquisition. Elution was accomplished by isocratic system of water:acetonitrile (15:85) with a flow rate of 10 mL/min. The Waters photodiode array (PDA) detector was set at 240 nm for the detection of eluted peaks. Abietic acid was eluted at 23.8 min (Figure 5A), was collected by a fraction collector, and was then dried under reduced pressure to yield about 13.5 mg.

#### 4.1.3. HPLC Analysis of Abietic Acid and Percent Purity

The purity of isolated abietic acid was determined by HPLC analysis. A solution of 1 mg/mL of purified abietic acid in MeOH was prepared and 10 μL were injected on a Waters Xterra RP-18 column (5 μm, 4.6 × 250 mm, Waters corporation, Dublin, Ireland). The column was eluted with an isocratic system of water: acetonitrile (1:9) with a flow rate of 1mL/min and the detection wavelength was set at 240 nm. The Empower^®^3 software package was implemented for spectral acquisition and data management. Abietic acid was eluted at 6.13 min (Figure 5B) and its percent purity was calculated in correlation to the percent area under each peak and was found to be 98.46%.

#### 4.1.4. MS Analysis of Abietic Acid

The purified abietic acid was dissolved in HPLC-grade methanol and analyzed using the single quadrupole Advion Compact Mass Spectrometer (Ithaca, NY, USA) with a detection mass range of 10–2000 Da (Figure 5C). The MS was equipped with an electrospray ionization (ESI) ion source and was operated in a negative mode (Figure 5D). The molecular ion peak [M-H]^−^ of abietic acid was detected at *m*/*z* 301.0 without correction. The CheMS Mass Express user interface (Advion, Ithaca, NY, USA) was used to operate the spectrometer, optimize the ion source, and control data acquisition parameters.

#### 4.1.5. Preparation *of P. paliustris* Total Extract, Abietic Acid and Standard Control Solutions

Pine extract was prepared as a stock solution of (50 mg/mL). Abietic acid, Raptinal, and Doxorubicin were prepared as stock solutions with 10mM concentration dissolved in DMSO (molecular biology grade from Sigma-Aldrich, St. Louis, MO, USA). The stock solutions were stored as aliquots at −20 °C in the dark. All reagents were molecular biology purity grade, obtained from (Sigma-Aldrich, St. Louis, MO, USA) unless otherwise specified in the corresponding sections.

### 4.2. In Vitro Study

#### 4.2.1. Cell Culture

All cell lines were purchased from an Egyptian company involved in the production of vaccines (VACSERA) and deposited in HSBR laboratory. Tested cancer cell lines included a human liver cancer cell line (HepG-2), a breast cancer cell line (MCF-7), and a human colon cancer cell line (HCT-116). The normal cell line used for comparison included fibroblast lung cells (WI-38). These cancerous cell lines were cultured in standard conditions using the corresponding documented media, including DMEM-high glucose for the cancerous cells (HepG-2, HCT-116 and MCF-7). EMEM media was used for normal cells (WI-38). All culture media were supplemented with 10% FCS as growth factor, 2m M-glutamine, and suitable units of penicillin and streptomycin (100 units from each). All cells were cultured at standard culture conditions, including temperature at 37 °C with 5% CO_2_. For quick splitting of cells, culture media were replaced every 2 days with 85–90% confluency after 3–4 days. Cell cultures were passaged using 0.25% trypsin/EDTA solution for downstream applications compatible with cell surface markers and cell membrane integrity.

#### 4.2.2. Viability Assay

Assessment of relative numbers of viable cells was performed using an MTT tetrazolium assay according to our previous protocol [90]. Briefly, pine extract, abietic acid, and standard positive controls (Raptinal and Doxorubicin) were tested in three independent experiments for calculation of IC_50_ after treatment for 50% reduction in growth. Pine extract was evaluated in cell lines at five dose concentrations (0.5, 0.05, 0.005, 0.0005, 0.00005 mg/mL in serial dilutions) while abietic acid and the positive controls were evaluated (100, 50, 0.1, 0.05 and 0.01 µM) for 24 h. Additionally, 0.1% DMSO-treated cells were used as negative control. The developed color, due to soluble formazan, was read at 570 nm with a microplate reader (800TSUV Biotek ELISA Reader, Agilent, Santa Clara, CA, USA), and corresponding optical densities were used for calculation of IC_50_. The corresponding selectivity index was calculated based on IC_50_ values of the pine extract and abietic acid on both noncancerous and cancerous cells according to Equation (1) [24,91]:

SI = IC_50_ (non-cancerous cells)/IC_50_ (cancerous cells) (1)

#### 4.2.3. Flow Cytometry and Apoptosis Assay

MCF-7 was the sensitive cancer cell line used for flow cytometric analysis at a density of 1 × 10^6^ cells per 25 cm^2^ flask for 24 h and treated with IC_50_ of pine extract, abietic acid and Raptinal positive control for 24 h. Cells were scraped gently and suspended in 50 μg /mL propidium iodide (PI) staining solution and 20 μg /mL RNaseA and incubated for 1h. Cell cycle analysis was performed using (Beckman Coulter Cytoflex, Indianapolis, Indiana, IN, USA) to detect cells in different cycle phases [92]. Fluorescence was measured on flow cytometer and the obtained cell histograms were analyzed. Annexin V-FITC apoptosis Detection ELISA kit was used for analysis of apoptosis. Apoptotic index (AI) is the parameter used to represent the efficacy of apoptosis after in vitro cell culture. It was calculated by dividing the percentage of early apoptotic cells (annexin+) by the total percentage of cells in the sample (apoptotic [annexin+] plus nonapoptotic cells [annexin-]) [93,94]. Similarly, G_2_M/G_0_-G_1_ ratio was calculated as % Gated cells in G_2_Mphase / % Gated cells in G_0_-G_1_ phase. The ratio was an indication of the proportion of dividing cells (cells in G_2_/M) to non-dividing cells [95,96]. As cells in G_2_/M will have 2x as much DNA as cells in G_0_/G_1_, they can be distinguished using simple DNA stains such as PI in flow cytometry.

#### 4.2.4. Real-Time Quantitative PCR (qPCR)

MCF-7 was also used in this assay for qPCR analysis of responded genes. Cells were seeded in 6-well plates at a density of 0.2 × 106 cells per well for 24 h after pine extract and abietic acid treatment, compared to 0.1% DMSO negative control cells. GeneJET RNA Purification spin column Kit (K0731, Thermo Fisher Scientific, Cairo, Egypt) was used for purification of RNA. High-Capacity cDNA Reverse Transcription Kit (4368814, Thermo Fisher Scientific, Cairo, Egypt) was used for cDNA synthesis. Quality and quantity of extracted RNA extraction and its cDNA copies were assessed using Nanodrop 2000C^®^ (Thermo Fisher Scientific, Cairo, Egypt) Optimization for used annealing temperatures of the primers, quantity of started cDNA and endogenous gene was performed before qPCR analysis with HERA SYBR^®^ Green qPCR Kit system (Willowfort, Birmingham, UK). GAPDH was selected as the endogenous gene and cDNA (3.02 ng/µL) as the constant starting concentration between different treated groups with calculated R2 = 0.97 (data not presented). Primer’s sequences of all selected genes are shown in Table S1.

#### 4.2.5. Immunocytochemistry

MCF-7 cells were seeded at 5000 cells on cover slips 22 × 22 mm (HUIDA, Yangzhou, China), with high precision (170 ± 5μm) in 6-well plates. MCF-7 cells were treated with both IC_50_ and 10-fold lower IC_50_ dose of pine extract and abietic acid for 24 h. At the end of the experiment, cells were fixed in 4% paraformaldehyde (PFA) in PBS for 30 min at room temperature and rinsed with PBS. For intracellular staining with both cytochrome-C in mitochondria and cytosolic release, 1% Triton-X-100 in PBS was used as permeabilization buffer for 10 min at room temperature. Nonspecific labelling was blocked by incubation for 1 h at room temperature with 1% bovine serum albumin in PBS with 0.2% Tween-20. Recombinant anti-cytochrome C antibody (Alexa Fluor^®^ 488) (ab192485, Thermo Fisher Scientific, Cairo, Egypt) was diluted (1:50) in blocking solution and incubated with cells overnight at 4 °C. After washing three times with PBS, nuclei were stained with Hoechst 33342 (Molecular Probes) at dilution 1:1000 in blocking solution and the coverslip was transferred in mounting media on standardized slides, then sealed with nail polisher. Fixed and stained cells were visualized using Carl Zeiss LSM 710 (Carl-Zeiss, Oberkochen, Germany) confocal microscopes. Each slide was scanned for different thicknesses and 10 µm was selected for maximum intensity of green staining (Supplementary Materials, scanning videos). Densitometric measurements of individual cells following immunocytochemical cytochrome-C were used to demonstrate the average intensity between different samples and each slide was scanned at eight different fields. Average intensity was calculated using ZEN 2.3 (Carl-Zeiss, Oberkochen, Germany). The fluorescent intensity levels within the areas of individual cells were obtained by detecting the outline of a cell and precured (Supplementary Materials, quantification images). For linearization and optimization of the experiment, whole cell measurements in different fields were taken, including nuclei, to ensure robust digitization each time cells were measured with their relative density within the nucleus and cytoplasm.

#### 4.2.6. Total Antioxidant Capacity

MCF-7 was used for assessment of antioxidant capacity of pine extract and abietic acid, compared to Doxorubicin, using commercial kits (Biodiagnostic, Giza, Egypt) with and without exposure to low conc. of H_2_O_2_ as an inducer of oxidative stress [97]. Briefly, MCF-7 were seeded in 6-well plates at a density of 0.2 × 106 cells per well for 12–24 h in standard conditions. A curve for the sensitivity of cell viability to H_2_O_2_ different concentrations (100, 50, 25, 12.5, and 6.25 µM) was constructed using MTT assay. The chosen concentration was 50 µM of H_2_O_2_. Subsequently, MCF-7 cells were seeded at a density of 0.2 × 10^6^ cells/well into 6-well plates under standard culture conditions. Then, two different independent experiments were performed for antioxidant calculation, as follows: (1) cells were treated with IC_50_ of pine extract, abietic acid, and Doxorubicin for 24 h, followed by lysis of cells, and total antioxidant induction was determined in cell lysate; (2) cells were exposed to 50 µM of H_2_O_2_ for 1 h, followed by treatment with IC_50_ of pine extract, abietic acid, and Doxorubicin for 24 h, followed by lysis of cells and determination of total antioxidant induction in cell lysate. The developed colored product was measured at 570 nm with a microplate reader (800TSUV Biotek ELISA Reader, Agilent, Santa Clara, CA, USA).

## 5. Conclusions

Breast cancer is one of the most prevalent types of invasive cancer in women. The treatment of breast cancer is obstructed by the adverse effects of existing chemotherapeutic agents, as well as the high progression of drug resistance. Plants serve as a pool of chemical entities that can assist in curbing various diseases, including cancer. Herein, pine extract and abietic acid, its naturally extracted pure compound, were tested for their anticancer potency against MCF-7 cancer cell line and a normal cell line, WI-38. We found that abietic acid showed dose-dependent growth inhibitory effects on the MF-7 cells. Abietic acid showed relatively lower cytotoxic effects over normal WI-38 cells. These observations agreed with the few available investigations in which abietic acid was studied, showing its suppressive effects on the growth and progression of some cancer cells. Moreover, it was found that abietic acid triggered total antioxidants in MCF-7 cells. This was accompanied by diminished intrinsic oxidative stress, which is responsible for carcinogenesis and resistance of MCF-7 cells. Since apoptosis is a vital anticancer target, used to eliminate cancer cells and maintain tissue homeostasis, we carried out annexin V/PI double staining of pine extract and abietic acid on treated-MCF-7 cells and found that abietic acid triggered cell cycle arrest in MCF-7 cells in G_2_/M phase as well as the SubG_0_-G_1_ subpopulation. Abietic acid-induced early-stage apoptosis was also accompanied by an upsurge of *caspases-3*, *caspase-8* and *Bax* and a decline in *Bcl-2*. Abietic acid could modulate gene expression of oncogenic genes such as c-myc, *TNF-α* and *NF-κB*. Abietic acid also downregulated the expression of many proliferation genes, such as *VEGF, TGF-β1* and *IGF1R*, that are known for their potential effects on angiogenesis, proliferation, metastasis, and invasion of MCF-7 cells. All these gene markers have been reported to be essential therapeutic biochemical targets for anticancer drugs. Cytosolic cytochrome-C protein expression release was observed, with significant increases in abietic acid-treated cells. Our recommendation for future studies on abietic acid would be to carry out an in vivo study using breast cancer animal models to confirm our hypothesis and suggest the appropriate sublethal dose for future clinical studies.

## Figures and Tables

**Table 1:** Antiproliferative activities of pine extract, abietic acid, Raptinal, and Doxorubicin against different cancerous cell lines (HepG-2, MCF-7 and HCT-116) compared to WI-38 normal cell line. SI (selectivity index) calculated as IC_50_ compound (WI-38)/ IC_50_ compound (cancer cell line).

Studied Plants & Reference Standard Drug	* IC_50_ ± SEM and Corresponding SI						
	Wi-38	HepG-2		MCF-7		HCT-116	
		** IC_50_	SI	** IC_50_	SI	** IC_50_	SI
Raptinal	5.2 ± 0.9	0.74 ± 0.1	7	4.1 ± 0.76	1.3	96.2 ± 1.5	0.05
Doxorubicin	0.1± 0.1	0.8 ± 1.1	0.11	1.3 ± 1.0	0.07	0.97 ± 0.72	0.1
Pine extract	0.42 ± 0.1	0.2 ± 0.76	2.1	0.11 ± 0.76	3.8	0.25 ± 0.1	1.7
Abietic acid	N/A	N/A	---	0.06 ± 0.5	>1	N/A	---

* IC_50_ of the pine extract was represented as (µg/mL) while IC_50_ of pure abietic acid, Raptinal, and Doxorubicin were represented as µM. ** IC_50_ Data represent mean ± SEM, *n* = 3. N/A (not applicable).

**Table 2:** Percentage of cell distribution in different cell cycle phases and subpopulations of MCF-7 cells using flow cytometry. MCF-7 treated with IC_50_ of pine extract and abietic acid and compared to both negative control (0.1% DMSO) and positive control (Raptinal). Pine extract and abietic acid significantly induce cell cycle arrest in different cell cycle phases. G_2_M/G_0_-G_1_ ratio, as marker of cell cycle arrest, was calculated for Raptinal-, pine extract-, and abietic acid-treated cells.

Cell Cycle Parameters on MF-7 Cells	Control	Raptinal	Abietic Acid	Pine Extract
% Gated Sub G_0_-G_1_ phase	1.52 ± 0.3	36.18 *** ± 6.2	20.96 *** ± 1.6	37.30 *** ± 7.5
% Gated G_0_-G_1_ phase	66.59 ± 4.8	33.62 ** ± 3.2	24.98 ** ± 5.2	42.07 * ± 9.1
% Gated S phase	13.16 ± 3.7	11.66 ± 1.8	4.96 * ± 1.04	9.29 * ± 1.5
% Gated G_2_M phase	18.73 ± 0.2	18.63 ± 3.8	49.17 ** ± 2.3	11.34 ± 2.3
G_2_M/G_0_-G_1_ ratio	0.28 ± 0.01	0.55 ± 0.1	2.0 ± 0.1	0.27 ± 0.1

Data represent mean ± SEM, *n* = 3. *p*-values for comparison with control non-treated cells is * *p* < 0.1, ** *p* < 0.01, *** *p* < 0.001.

**Table 3:** Percentage of viable, apoptotic, late apoptotic, and necrotic MCF-7 cells using flow cytometry. MCF-7 were treated with IC_50_ of pine extract and abietic acid and compared to both negative control (0.1% DMSO) and positive control (Raptinal). Apoptotic index, as marker of apoptosis, was calculated for Raptinal-, pine extract-, and abietic acid-treated cells.

Apoptotic Parameters on MF-7 Cells	Control	Raptinal	Abietic Acid	Pine Extract
% Viable cells (C^− −^)	93.65 ± 10.7	48.55 ** ± 4.8	8.48 *** ± 2.9	28.55 *** ± 10.9
% Early apoptotic cells (C^+^^−^)	6.30 ± 2.4	51.35 *** ± 11.5	91.41 *** ± 12.9	71.39 *** ± 7.6
% Late apoptotic cells (C^− +^)	0.01	0.00	0.01	0.00
% Necrotic cells (C^+ +^)	0.04	0.1	0.1	0.06
Apoptotic index (AI)	0.06 ± 0.03	0.51 ± 0.4	1.0 ± 0.35	0.71 ± 0.11

Data represent mean ± SEM, *n* = 3. *p*-values for comparison with control nontreated cells are ** *p* < 0.01, *** *p* < 0.001.

**Table 4:** Temporal gene expression analysis of selected genes at early stage, expressed as *x*-fold change for MCF-7 cells treated with IC_50_ of pine extract and abietic acid at 4, 8, and 24 h, compared to negative control cells treated with 0.1% DMSO. Data represent mean ± SEM, *n* = 3. Downregulated genes have RQ values less than 1 with negligible SEM.

Genes	Pine Total Extract-RQ, Fold-Change (Mean ± SEM)			Abietic Acid-RQ, Fold-Change (Mean ± SEM)		
	4 h	8 h	24 h	4 h	8 h	24 h
**Apoptotic genes**						
*Fas*	145.0 ± 10.2	308.0 ± 5.7	146.0 ± 6.4	7.0 ± 0.9	32.0 ± 2.7	33.0 ± 8.7
*FasL*	26.0 ± 2.3	319.0 ± 8.7	88.0 ± 12.5	10.0 ± 1.4	27 ± 5.4	5.0 ± 1.8
*BINP3*	0.15	57.0 ± 6.7	5.0 ± 2.6	7.0 ± 2.3	16.0 ± 4.7	0.22
*Casp3*	1.8 ± 0.2	28.0 ± 5.2	19.0 ± 6.9	0.009	0.02	1.1 ± 0.1
*Casp8*	2794 ± 15.7	1285.0 ± 51.9	37.0 ± 4.2	42.0 ± 5.2	7.0 ± 4.9	3.0 ± 0.7
*Cyt-C*	3712 ± 20.8	5036.0 ± 44.2	1009.0 ± 10.4	4.0 ± 1.4	772.0 ± 13.8	2688.0 ± 17.5
*Bax*	0.01	0.07	2.0 ± 0.5	2.0 ± 0.7	5.0 ± 1.2	0.05
*Bcl-2*	10 ± 1.7	8.0 ± 2.8	3.0 ± 0.4	5.0 ± 1.8	3.0 ± 1.1	0.36
*Bax/Bcl-2 ratio*	0.01	0.01	0.7	0.01	0.7	13.9 ± 1.4
*ATG5*	0.45	1.1 ± 0.1	6.0 ± 1.7	0.14	0.88	1.5 ± 0.4
*P53*	0.45	0.13	0.53	0.06	0.81	2.0 ± 0.6
**Proliferation genes**						
*VEGF*	0.45	0.52	0.03	3.0 ± 2.7	4.0 ± 1.3	0.03
*TGF-β1*	0.25	0.09	0.01	0.77	0.57	0.02
*IGF1R*	0.32	0.99	0.80	2.0 ± 0.7	1.3 ± 0.2	0.04
*ATG12*	0.20	0.28	0.22	0.12	0.36	0.03
**Oncogenic genes**						
*C-myc*	0.16	0.15	0.03	0.18	0.29	0.05
*TNF-α*	2.0 ± 0.5	4.0 ± 0.9	4.0 ± 0.7	0.02	0.04	0.01
*NF-κB*	0.03	0.44	0.17	1.4 ± 0.2	2.6 ± 0.7	1.0 ± 0.1
**Kinase’s genes**						
*PKC-α*	21.0 ± 8.7	2.0 ± 0.7	0.88	3.0 ± 0.9	2.0 ± 0.4	0.30
*PRKAA1*	0.49	42.0 ± 16.7	50.0 ± 7.8	0.77	0.5	7.0 ± 2.4
*CDK-4*	0.1	1.6 ± 0.3	0.47	9.0 ± 2.6	6.0 ± 1.5	0.1

## Data Availability

The data presented in this study are available in this article.

## Supplementary Materials

The following are available online, Figure S1: Sigmoidal dose-response relation for calculation of IC_50_ of (A) Pine extract and (B) Abietic acid on HepG-2, MCF-7 and HCT-11 cancerous cell lines and WI-38 normal cells. Table S1: Primer’s sequences of apoptotic genes used for gene expression analysis, Video S1: Scanning video for immunocytochemistry fluorescence calculation of abietic acid.

## Sample Availability

Samples of the pine extract/abietic acid are available from the authors upon request.
