# Supplementary material for: Growth Inhibition and Apoptotic Effect of Pine Extract and Abietic Acid on MCF-7 Breast Cancer Cells via Alteration of Multiple Gene Expressions Using In Vitro Approach
Source: Molecules. 2022 Jan 4;27(1):293. doi: 10.3390/molecules27010293 (PMC8746537; doi:10.3390/molecules27010293)
Supplement: Supplementary file 1 [file molecules-27-00293-s001.zip › molecules-1444345 - Supplementary materials.pdf]

Supplementary Materials

Growth Inhibition and Apoptotic Effect of Pine Extract and Abietic Acid on MCF-7 Breast Cancer Cells via Alteration of Multiple Gene Expressions using *In-Vitro* Approach

Hesham Haffez [1,2*], Shimaa Osman [3], Hassan Ebrahim [4] and Zeinab Hassan [1]

[1] Biochemistry and Molecular Biology Department, Faculty of Pharmacy, Helwan University, P.O. Box 11795,Cairo,Egypt; shaimaa_abdelrheem@hotmail.com
[2] Helwan Structural Biology Center for Excellence, Helwan University, P.O. Box, 11795, Cairo, Egypt
[3] Helwan General Hospital, P.O. Box 11731, Cairo, Egypt; shaimaa_abdelrheem@hotmail.com
[4] Pharmacognosy Department, Faculty of Pharmacy, Helwan University, P.O. Box 11795,Cairo, Egypt; hebrahim@pharm.helwan.edu.eg
* Correspondence: author, E-mail address: hesham.haffez@pharm.helwan.edu.eg, Tel.: +201094970173

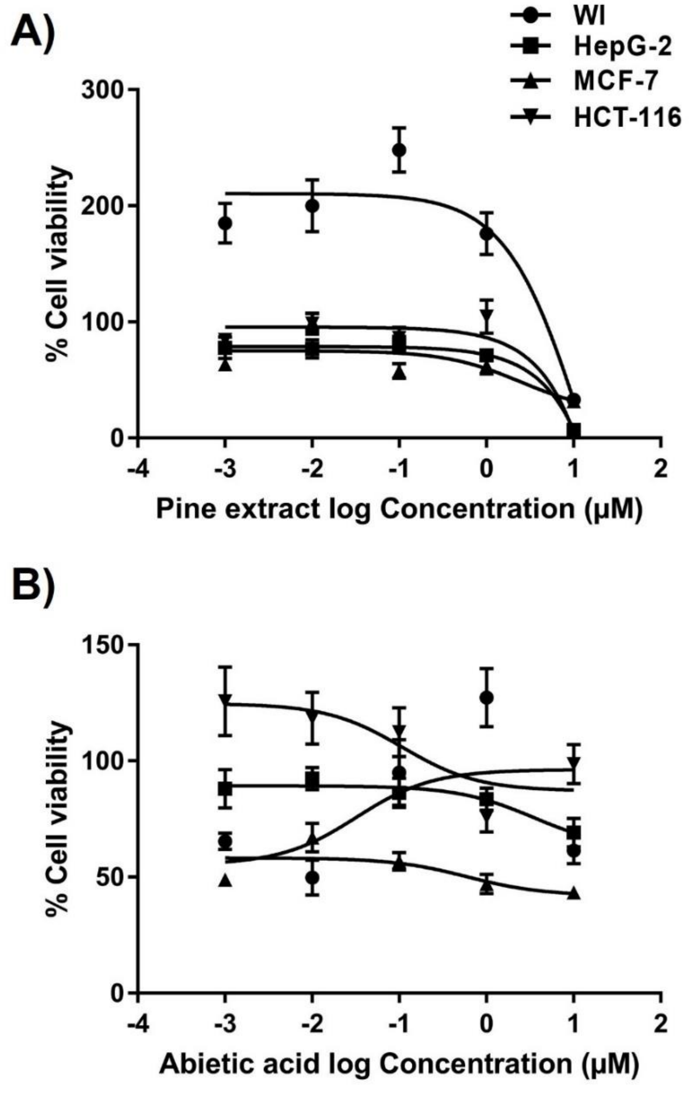

Figure S1. Sigmoidal dose-response relation for calculation of IC_{50} of **A**) Pine extract and **B**) Abietic acid on HepG-2, MCF-7 and HCT-11 cancerous cell lines and WI-38 normal cells.

Table S1. Primer's sequence of apoptotic genes used for gene expression analysis.

Gene	Forward primer sequence	reverse primer sequence
Fas	5'- CAAGTGACTGACATCAACTCC -3'	5'- CCTTGGTTTTCCTTTCTGTGC -3'
FasL	5'- ACACCTATGGAATTGTCCTGC -3'	5'- GACCAGAGAGAGCTCAGATACG -3'
BNIP3	5'- GCTCCCAGACACCACAAGAT -3'	5'- TGAGAGTAGCTGTGCGCTTC -3'
Cas3	5'- ACATGGAAGCGAATCAATGGACTC -3'	5'- AAGGACTCAAATTCTGTTGCCACC -3'
Cas8	5'- AGAGTCTGTGCCCAAATCAAC -3'	5'- GCTGCTTCTCTCTTTGCTGAA -3'
Cyto C	5'- GAGGCAAGCATAAGACTGGA -3'	5'- TACTCCATCAGGGTATCCTC -3'
Bax	5'- CCCGAGAGGTCTTTTTCCGAG -3'	5'- CCAGCCCATGATGGTTCTGAT -3'
Bcl2	5'- TTGTGGCCTTCTTTGAGTTCGGTG -3'	5'- GGTGCCGGTTCAGGTACTCAGTCA -3'
ATG5	5'- TTTTCACTGTGGTCCCTGGC -3'	5'- ATCCCCAAAATGAACCGACG -3'
p53	5'- GCCCAACAACACCAGCTCCT -3'	5'- CCTGGGCATCCTTGAGTTCC -3'
VEGF	5'- GCAGACCAAAGAAAGATAGAGCAAG -3'	5'- CGCCTCGGCTTGTCACAT -3'
TGF-β1	5'- CACCCGCGTGCTAATGG -3'	5'- ATGCTGTGTGTACTCTGCTTGAACT -3'
IGF1R	5'- CTCCTGTTTCTCTCCGCCG -3'	5'- ATAGTCGTTGCGGATGTCGAT -3'
ATG12	5'- TAGAGCGAACACGAACCATCC-3'	5'- CACTGCCAAAACACTCATAGAGA -3'
C-myc	5'- AAACACAAACTTGAACAGCTAC -3'	5'- ATTTGAGGCAGTTTACATTATGG -3'
TNF-α	5'- ATGAGCACTGAAAGCATGATCC -3'	5'- GAGGGCTGATTAGAGAGAGGTC -3'
NF-κB	5'- ATGGCTTCTATGAGGCTGAG -3'	5'- GTTGTTGTTGGTCTGGATGC -3'
C-myc	5'- AAACACAAACTTGAACAGCTAC -3'	5'- ATTTGAGGCAGTTTACATTATGG -3'
PKC-α	5'- CCTATGGCGTCCTGTTGTAT -3'	5'- GTTTGTTCTCGCTGGTGAGT -3'
PRKAA1	5'- GCTCACCCAACTATGCTGCAC -3'	5'- TATCTACCTCTGGGCCTGCATACAA -3'
CDK-4	5'- CTGGTGTTTGAGCATGTAGACC -3'	5'- AAACTGGCGCATCAGATCCTT -3'
GAPDH	5'- CTGACTTCAACAGCGACACC -3'	5'- TAGCCAAATTCGTTGTCATACC -3'
